# Arecoline inhibits the growth of 3T3-L1 preadipocytes via AMP-activated protein kinase and reactive oxygen species pathways

**DOI:** 10.1371/journal.pone.0200508

**Published:** 2018-07-16

**Authors:** Zi-Han Tian, Jueng-Tsueng Weng, Li-Jane Shih, An-Ci Siao, Tsai-Yun Chan, Yi-Wei Tsuei, Yow-Chii Kuo, Tsu-Shing Wang, Yung-Hsi Kao

**Affiliations:** 1 Department of Life Sciences, National Central University, Jhongli, Taoyuan, Taiwan; 2 Chung Shan Hospital, Taipei, Taiwan; 3 Medical Laboratory, Taoyuan Armed Forces General Hospital, Taoyuan City, Taiwan; 4 National Defense Medical Center, Taipei, Taiwan; 5 Department of Emergency, Taoyuan Armed Forces General Hospital, Taoyuan City, Taiwan; 6 Department of Gastroenterology, Taiwan Landseed Hospital, Taoyuan City, Taiwan; 7 Department of Biomedical Sciences, Chung Shan Medical University, Taichung, Taiwan; National Health Research Institutes, TAIWAN

## Abstract

The present study was designed to investigate the pathways involved in the effect of betel nut arecoline on cell viability in 3T3-L1 preadipocytes. Arecoline, but not arecaidine or guvacine, inhibited preadipocyte viability in a concentration- and time-dependent manner. Arecoline arrested preadipocyte growth in the G2/M phase of the cell cycle; decreased the total levels of cyclin-dependent kinase 1 (CDK1), p21, and p27 proteins; increased p53 and cyclin B1 protein levels; and had no effect on CDK2 protein levels. These results suggested that arecoline selectively affected a particular CDK subfamily. Arecoline inhibited AMP-activated protein kinase (AMPK) activity; conversely, the AMPK activator, AICAR, blocked the arecoline-induced inhibition of cell viability. Pre-treatment with the antioxidant, *N*-acetylcysteine, prevented the actions of arecoline on cell viability, G2/M growth arrest, reactive oxygen species (ROS) production, and the levels of CDK1, p21, p27, p53, cyclin B1, and phospho-AMPK proteins. These AMPK- and ROS-dependent effects of arecoline on preadipocyte growth may be related to the mechanism underlying the modulatory effect of arecoline on body weight.

## Introduction

Obesity can be characterized as an increase in adipocyte lipid content, due to differentiation, and an increased number of fat cells, due to mitogenesis [1]. These two cellular processes can be regulated by dietary factors [2]. Betel nut alkaloids (BNAs), particularly arecoline, have been described as regulatory agents for obesity and modulators of fat cell adipogenesis [3–9]. Some *in vivo* studies have shown that consumption of betel quid or areca nut was associated with obesity and metabolic syndrome [3–4, 6–9]. However, that association remains to be demonstrated clinically. Others found a negative relationship between betel nut chewing and body weight from laboratory animal studies [5,8]. These different links between betel nut consumption and body weight from epidemiological studies and animal studies may indicate that betel nut has diverse functional effects in different species or systems. Thus, controversy has arisen about the direct effects of BNAs on fat cells. One potential explanation for the disparate findings might be that betel nut may harbor various alkaloids with different effects on the signaling cascades in fat cells. Accordingly, careful examination of how BNAs are involved in the direct regulation of fat cell growth may improve our understanding of the relationship between arecoline and body weight.

*In vitro* studies have shown that arecoline inhibited lipid accumulation. In particular, arecoline blocked insulin signaling and glucose uptake in 3T3-L1 adipocytes. In addition, arecoline reduced lipid storage, inhibited fatty acid synthase (a lipogenic enzyme) expression [10–11], and stimulated lipolysis in adipocytes [11]. Although arecoline had various biological effects on adipocytes [10–12], no studies have demonstrated whether arecoline or other BNAs affected preadipocytes. In non-fat cells, arecoline could induce dysregulation of the G1 or G2 growth phase of the cell cycle by modulating the expression of cell cycle-related proteins (e.g., p21 and cyclin-dependent kinases [CDKs]) and altering the production of reactive oxygen species (ROS) [13–16]. However, it remains unknown whether arecoline or other BNAs can alter the cell cycle in preadipocytes. To elucidate the mechanisms that underlie the actions of arecoline and other BNAs on fat cells, it might be useful to determine their effects on cell cycle-control proteins and ROS production in preadipocytes.

In the present study, we investigated the mechanism by which betel nut arecoline inhibited cell viability in 3T3-L1 preadipocytes, and we compared the effects to those of arecaidine and guvacine. First, we showed that arecoline, but not arecaidine or guvacine, significantly reduced preadipocyte viability. We found that arecoline, but not the other two BNAs, induced dysregulation of the cell cycle in preadipocytes; in addition, we observed significant changes in the levels of cell cycle-related proteins. We also showed that arecoline-regulated preadipocyte viability depended on intracellular ROS production and the inhibition of AMP-activated protein kinase (AMPK). Moreover, arecoline, but not arecaidine or guvacine, significantly reduced total triglyceride accumulation during adipogenic differentiation.

## Methods

### Chemical reagents

All reagents (e.g., arecoline hydrobromide, arecaidine hydrochloride, guvacine hydrochloride, etc.) were obtained from Sigma Chemical (St. Louis, MO), unless otherwise stated. BNAs were dissolved in sterile medium for cell treatment. As described in detail previously [17], DMEM, calf serum (CS), trypsin, and protein markers were purchased from Gibco-Invitrogen (Grand Island, NY). Antibodies specific for AMPK, phospho-AMPK, and cyclin B1 were obtained from Cell Signaling Technology (Billerica, MA, USA). All other antibodies (i.e., anti-CDK1, anti-CDK2, anti-p21, anti-p27, anti-p53, anti-actin, donkey anti-rabbit IgG-HRP, etc.) were purchased from Santa Cruz Biotechnology (Santa Cruz, CA).

### Cell culture

As described in detail previously [1], 3T3-L1 cells (American Type Culture Collection, Manassas, VA) were grown at a density of 15,000~20,000 cells/cm^2^ in DMEM (pH 7.4) containing 10% CS, 100 units/ml of penicillin, and 100 μg/ml streptomycin (GibcoBRL) in a humidified atmosphere of 95% air and 5% CO_2_ at 37°C. The medium (10 ml) was replaced every 2 days. Serum components contained factors that facilitated 3T3-L1 differentiation from preadipocytes to adipocytes when they reached confluency; therefore, we subcultured the cells before they reached confluency.

### Cell viability

3T3-L1 cells (6000 cells/well) were seeded in triplicate wells of a 96-well plate [18]. To determine whether BNAs had a dose- or time-dependent effect on the viability of 3T3-L1 preadipocytes, we treated cells with arecoline, arecaidine, or guvacine, at various concentrations (0~1000 μM) in the presence of 10% CS-supplemented medium for the indicated time periods. Then, we determined optimal conditions for arecoline modulation of 3T3-L1 preadipocyte viability. After incubating cells for the indicated times, we added tetrazolium dye, MTT (3-(4,5-dimethylthiazol-2-yl)-2,5-diphenyltetrazlium bromide), and incubated cells in the dark at 37°C for 3 h. Next, the medium was removed, and we added 100 μl of 100% DMSO in each well to stop the reaction. Then, cells were incubated for 10 min on a shaker at 300 rpm. The insoluble product (formazan) dissolved in the DMSO, and the absorbance was read at 570 nm.

### Experimental treatment

We compared the effects of different BNAs on the different phases of the cell cycle, on the levels of cell cycle-related proteins, and on ROS production. 3T3-L1 cells were treated with 400 μM arecoline, arecaidine, or guvacine for 24 or 48 h. After incubation, we determined changes in levels of the factors of interest.

To investigate whether altering AMPK activity affected the arecoline-mediated viability of 3T3-L1 preadipocytes, we followed the methods reported by Ku et al. (2014) [19]. Briefly, cells were pretreated for 1 h with either the AMPK activator, N^1^-(β-D-ribofuranosyl)-5-aminoimidazole-4-carboxamide (AICAR; TOCRIS Bioscience, Bristol, UK; 0~250 μM), or the AMPK inhibitor, compound C (10 μM). These compounds were dissolved in 100% DMSO, and added to the culture medium at a final concentration of 0.1%. Next, cells were treated with 400 μM arecoline for 24 or 48 h, and cell viability was measured.

We studied the ROS-dependent effect of arecoline and other BNAs on preadipocyte viability with the methods described by Wang et al. (2009) [20]. Briefly, 3T3-L1 preadipocytes were pretreated with the antioxidant, *N*-acetylcysteine (NAC; 0~5 mM), for 1 h. Then, cells were exposed to different doses of arecoline, arecaidine, or guvacine. After 24 or 48 h, we measured cell viability, the percentage of cells in the different phases of the cell cycle, levels of cell cycle-related proteins, and levels of ROS production.

### Flow cytometric analysis

Changes in the kinetics of the cell cycle were analyzed with flow cytometry, as described in detail previously by Hung et al. (2005) [1]. Briefly, 3T3-L1 cells (6 × 10^5^ cells/plate) were plated in a 10-cm dish containing 10 ml DMEM supplemented with 10% CS. After one day in culture, cells were treated with fresh medium containing 10% CS with or without various concentrations of arecoline, arecaidine, or guvacine, for different time periods. The harvested cell pellets were fixed in 70% ethanol and stored at -20°C until later analysis. For analysis, cell pellets were washed with 10 mM cold PBS (pH 7.4), incubated at 37°C for 30 min with 100 μg/ml RNase A, and then stained with 200 μg/ml propidium iodide in PBS containing 1% Triton X-100. Cell cycle profiles and distributions were determined with flow cytometric analyses of 10^4^ cells, performed with the CELLQuest program on a FACS Calibur flow cytometer (Becton-Dickinson, San Jose, CA). Clumped cells were excluded from the cell cycle distribution analysis with gating.

### Western blot analysis

Western blot analyses were performed with supernatant fractions of preadipocytes, as described in detail previously by Hung et al. (2005) and Ku et al. (2009) [1,17]. An aliquot of 50~75 μg of supernatant protein was separated on a 12% SDS-PAGE, prepared with 2× gel-loading buffer (100 mM Tris-HCl, pH 6.8, 4% SDS, 20% glycerol, 0.2% bromophenol blue, and 10% β-mercaptoethanol). The separated proteins were then blotted onto Immobilon-NC transfer membranes (Millipore, Bedford, MA) and blocked for 1 h at room temperature with 10 mM PBS containing 0.1% Tween 20 (PBST) and 5% nonfat milk. After washing with PBST, primary antibodies (e.g., anti-CDK1, anti-CDK2, anti-p21, anti-p27, anti-p53, anti-cyclin B1, and actin antisera) were added at a dilution of 1:1000 (~0.2 μg/ml) and incubated overnight at 4°C. After washing with PBST, the following secondary antibodies were added: donkey anti-rabbit IgG, donkey anti-goat IgG, and goat anti-mouse IgG, conjugated with horseradish peroxidase, all diluted at 1:2000 (~0.2 μg/ml). The immunoblots were visualized by adding the Western Lightning^™^ Chemiluminescence Reagent Plus (Perkin-Elmer Life Science, Boston, MA) for 3 min, followed by exposure to Fuji film for 2~3 min. Blots were quantified with the FX Pro Plus Molecular Imager^®^ (Bio-Rad Laboratories, Hercules, CA). After normalization to β-actin protein, the levels of these intracellular proteins were expressed as a percent of the control, unless noted otherwise.

### Reactive oxygen species (ROS)

We performed ROS assays according to the methods described by Wang et al. (2009) [20]. Briefly, 3T3-L1 cells (1.2 × 10^5^ cells/well) were plated in a 6-well plate containing 10 ml DMEM supplemented with 10% CS. After one day in culture, cells were treated with fresh medium containing 10% CS, with or without various concentrations of arecoline, arecaidine, or guvacine, for various time periods. After treatment, cells were washed with 10 mM PBS (pH 7.4); then, 30 μM of 2’,7’-dichlorofluorescein diacetate (DCFDA) was added. After a 30-min incubation in the dark at 37°C, cells were washed with PBS to remove free DCFDA from the medium. Dichlorofluorescein was produced from the reaction of DCFDA with ROS. We detected this fluorescence signal with a fluorescence spectrophotometer (F-4500, HITACHI); the excitation wavelength was 504 nm and the emission wavelength was 529 nm. The absorbance values were normalized to the number of cells; then, the ROS level was expressed as the signal intensity as a multiple of the control signal intensity. Cells were trypsinized and counted with the 0.4% trypan blue exclusion method. Only live cells are represented in this study.

### Statistical analysis

We expressed all data as the mean ± SEM. The statistical analysis was performed as described previously by Ku et al. (2012) [21].

## Results and discussion

### Preadipocyte viability inhibited by particular betel nut alkaloids

**Fig 1:**
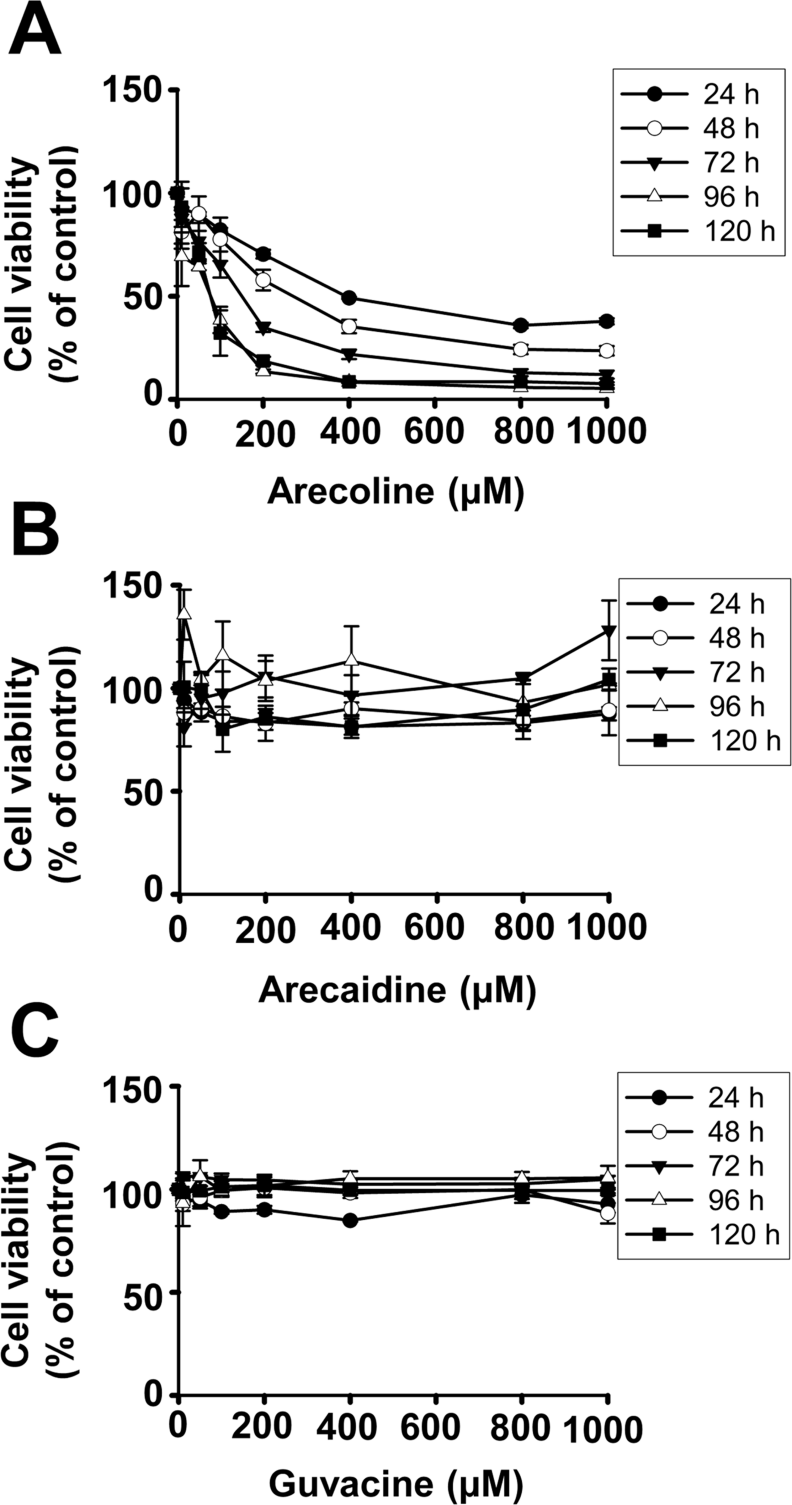
Reductive effect of betel nut alkaloids on cell viability of 3T3-L1 preadipocytes depending on the dosage (0~1,000 μM) and duration (24~120 h) of treatment, and on the types of alkaloids. Data are expressed as the mean ± SEM from triplicate determinations. For clarity, statistical significances are not shown.

Cell viability varied significantly when 3T3-L1 preadipocytes were incubated with different concentrations of arecoline, arecaidine, or guvacine (Fig 1). In general, arecoline (Fig 1A) reduced cell viability more effectively than arecaidine (Fig 1B) or guvacine (Fig 1C). The effects depended on the dosage and duration of treatment. For example, the IC_50_ values of arecaidine and guvacine to preadipocytes were greater than 1000 μM during the 72 h of treatment. The IC_50_ values of arecoline were 200~400 μM at 24 and 48 h; however, they were below 200 μM at 72~120 h.

### Preadipocyte growth arrest induced by betel nut arecoline

**Fig 2:**
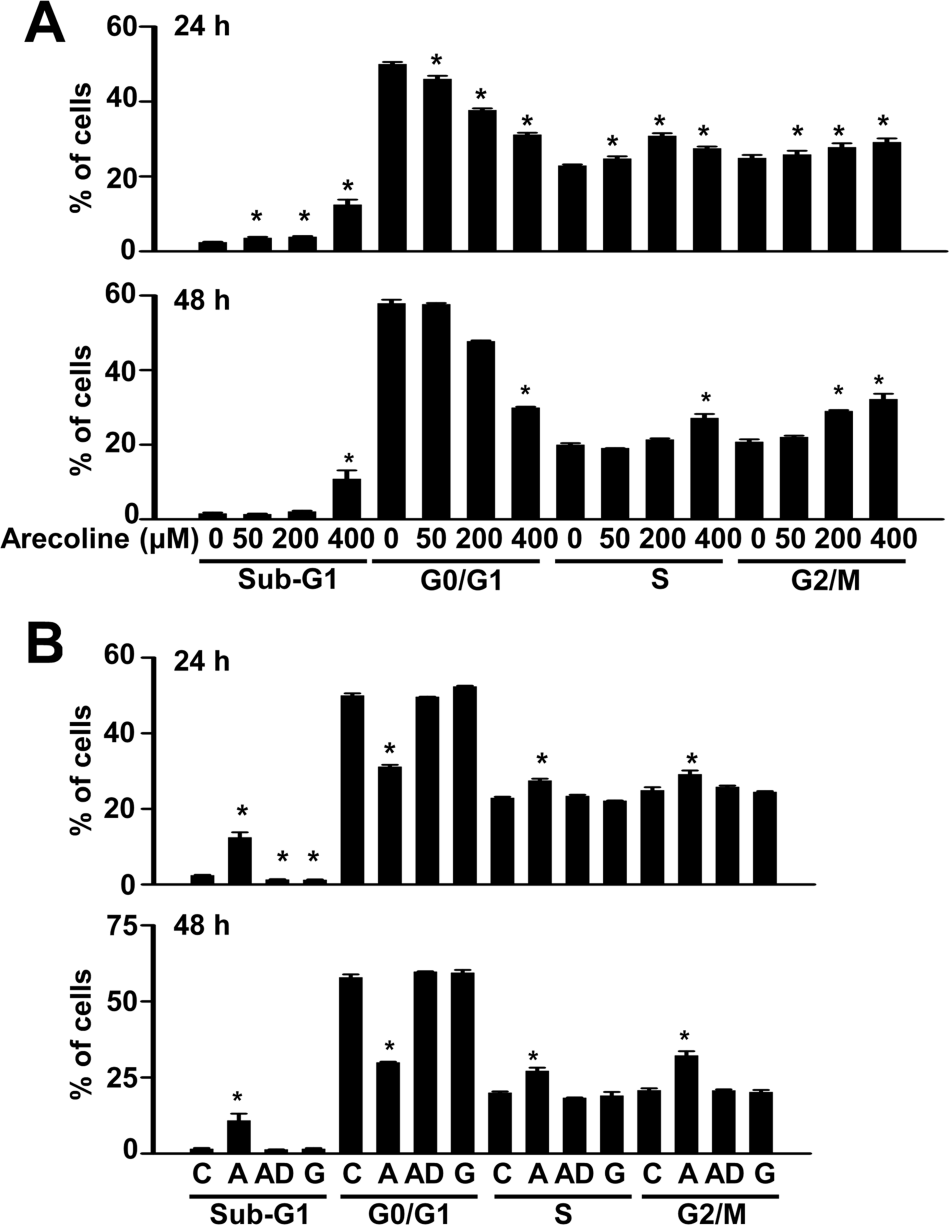
Effects of betel nut arecoline, but not arecaidine or guvacine, on the different phases of the cell cycle in 3T3-L1 preadipocytes. (A) A dose-dependent effect of arecoline on the cell cycle, as examined by flow cytometry, was observed after 24 and 48 h of treatment. (B) An alkaloid-specific effect of betel nut on the cell cycle was observed after 24 and 48 h of 400-μM treatment. Data are expressed as the mean ± SEM of triplicate determinations. C, control; A, arecoline; AD, arecaidine; G, guvacine. The asterisk indicates the significance of the difference (*p* < 0.05) from the control in a given phase of the cell cycle. In some groups, standard error bars are too small to be seen.

Cell growth can be controlled by alterations in the cell cycle [1,13–16,22]. Accordingly, we investigated whether arecoline induced alterations in the phases of the preadipocyte cell cycle (Fig 2). The sub-G0, G0/G1, S, and G2/M phases of the preadipocyte cell cycle were analyzed separately with flow cytometry. We found that arecoline altered the percentages of cells in the different the cell cycle phases of 3T3-L1 preadipocytes in a dose- and time-dependent manner (Fig 2). Generally, arecoline at 200~400 μM for 24 or 48 h tended to increase the percentages of cells in sub-G0, S, and G2/M phases and reduced the percentage of cells in the G0/G1 phase (Fig 2A). At 400 μM, neither arecaidine nor guvacine altered the percentages of cells in the G0/G1, S, or G2/M phases of the 3T3-L1 preadipocyte cycle, compared to the control (Fig 2B). These observations suggested that the effect of betel nut on the cell cycle of preadipocytes was alkaloid-specific. Thus, arecoline might reduce 3T3-L1 preadipocyte viability by inducing dysregulation of the cell cycle, as suggested by the reduction in G1 growth arrest and the increases in S phase and G2/M growth arrest. This interpretation was consistent with previous reports that described a G2/M-dependent effect of arecoline on mucosal fibroblasts, vascular endothelial cells, and basal cell carcinoma cells [13,15,23]. However, it was inconsistent with other reports that described G0/G1-dependent effects of arecoline on normal hepatocytes and keratinocytes [14,15]. Because arecoline also increased the percentage of 3T3-L1 preadipocytes in the sub-G0 phase, an indicator of cell apoptosis, we could not exclude the possibility that arecoline might have reduced 3T3-L1 preadipocyte viability through an apoptotic effect, consistent with reports of arecoline effects on human basal cell carcinoma cells and oral fibroblasts [23–24].

### Arecoline selectively affected some cell cycle-regulating proteins

**Fig 3:**
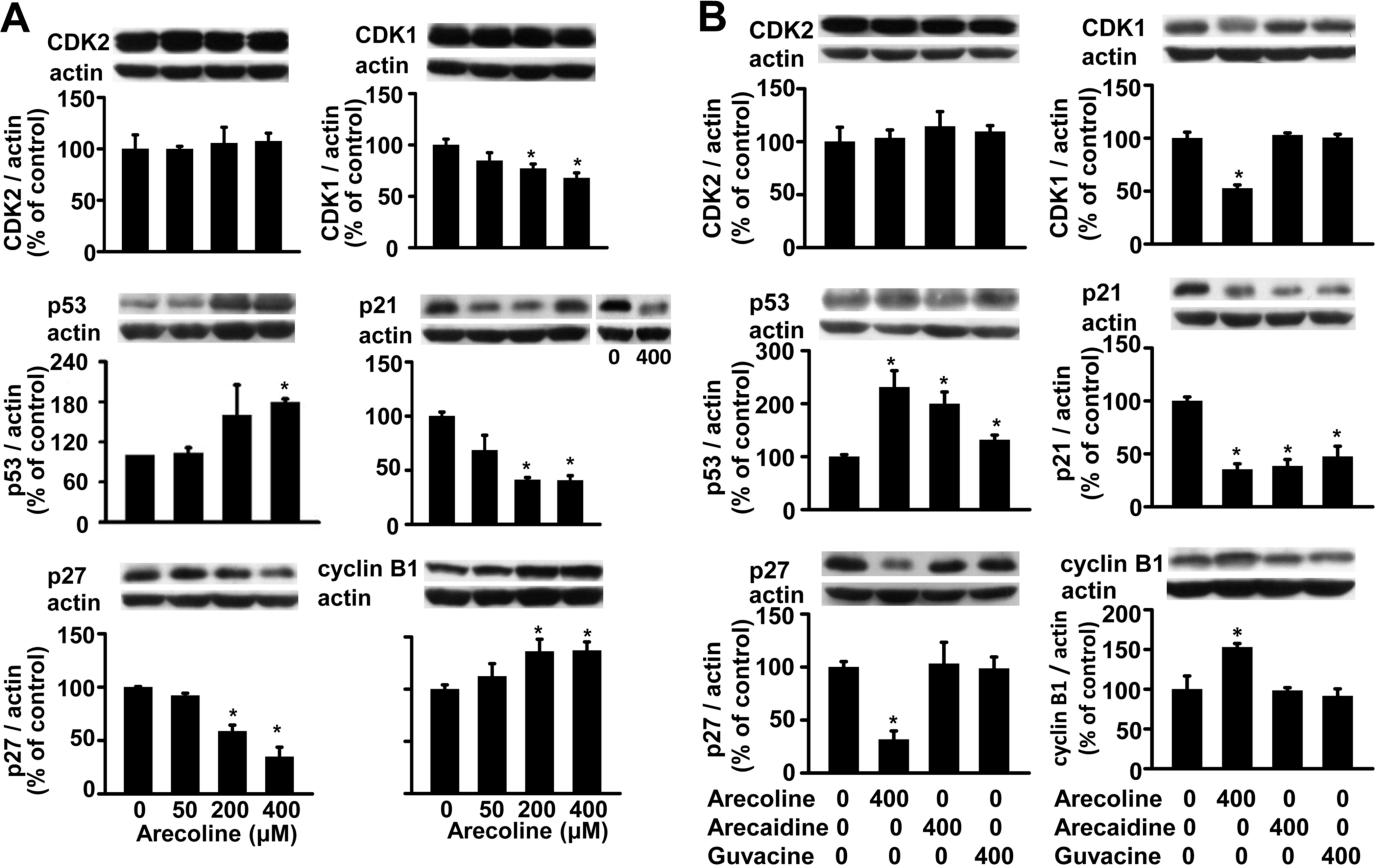
Effects of betel nut alkaloids on protein amounts of the cell cycle-controlling pathway proteins, such as CDK2, CDK1, p53, p21, p27, and cyclin B1, in 3T3-L1 preadipocytes. (A) A dose-dependent effect of arecoline was observed after 24 h of treatment. (B) An alkaloid-specific effect of betel nut was observed after 24 h of 400-μM treatment. These proteins were measured by Western blot analysis and then expressed after normalization to actin. In (A), we added an additional Western blot of p21 protein to the right side to indicate its significant decrease after 24 h of 400-μM arecoline treatment when compared to the control. Data are expressed as the mean ± SEM from triplicate determinations; each determination was pooled from four 10-cm culture plates. *, *p* < 0.05 *vs*. the control.

The preadipocyte cell cycle can be regulated by the CDK family, p21, p27, p53, and cyclin [25]. Previous studies showed that some of these proteins were targeted by arecoline to mediate changes in the cell cycle of non-fat cells [13–16]. Generally, CDK1 and CDK2 control the checkpoints of the G1 phase and the G2/M phase, respectively. Both p21 and p27 act as endogenous inhibitors of CDK. Cyclin B1 acts as an endogenous activator of CDK1. We investigated which signaling molecules arecoline acted on to alter the 3T3-L1 preadipocyte cell cycle. We examined whether 24 h of arecoline affected the levels of CDK1, CDK2, p21, p27, p53, and cyclin B1 proteins (Fig 3). We found that arecoline did not alter CDK2 protein levels, but it reduced CDK1 protein expression (Fig 3A). The effect of arecoline was dose-dependent. At 200 and 400 μM arecoline, the total levels of p53 and cyclin B1 proteins were significantly increased, and the levels of p21 and p27 were reduced. Interestingly, arecaidine and guvacine (400 μM for 24 h) did not alter the total protein levels of CDK1, CDK2, p27, or cyclin B1, but they significantly increased the p53 protein level and decreased the p21 protein level (Fig 3B).

Previous studies have shown that a variety of signaling proteins are involved in arecoline effects on cell growth, in non-fat cells, and on glucose uptake, in 3T3-L1 preadipocytes [1,10–16,23–24]. To our knowledge, CDKs and their related inhibitors and activators are key regulators of the cell cycle in 3T3-L1 cells. These molecules mediate the effects of green tea catechins on growth arrest and apoptosis in preadipocytes [1,18,25]. We studied these proteins to elucidate the mechanism underlying arecoline alterations in the 3T3-L1 preadipocyte cell cycle. We observed that 24 h of 200~400 μM arecoline did not alter CDK2 protein levels. We evaluated p21 and p27 levels, because they are endogenous inhibitors of CDK2, and they can induce cell cycle arrest at the G1 checkpoint [22,26–28]. We found that 24 h arecoline reduced p21 and p27 protein expression. That finding suggested that arecoline had indirect effects on CDK2, which might lead to the significant reductions observed in the percentages of cells in the G0/G1 phase and the increases observed in the percentages of cells in the S and G2/M phases after 24 and 48 h treatments. It has been shown that CDK1 takes over as the predominant CDK regulator in the early G2/M transition of the cell cycle [22,26–28]. Therefore, the observed decrease in CDK1 protein expression by 200~400 μM arecoline suggested that arecoline acted on the G2/M phase of preadipocytes to induce cell cycle arrest at the G2/M checkpoint. Cyclin B1 is a G2/M cyclin associated with CDK1, and its accumulation favors M-phase arrest at the G2/M checkpoint [22,29]. Accordingly, our finding that 200~400 μM arecoline for 24 h increased cyclin B1 protein expression also strengthened the interpretation that arecoline action on CDK1 induced preadipocyte growth arrest at the G2/M phase. These findings were consistent with a previous study that reported arecoline induction of G2/M-phase arrest in KB human epidermoid carcinoma cells [29,30].

### Arecoline effect on preadipocyte growth depended on AMPK

**Fig 4:**
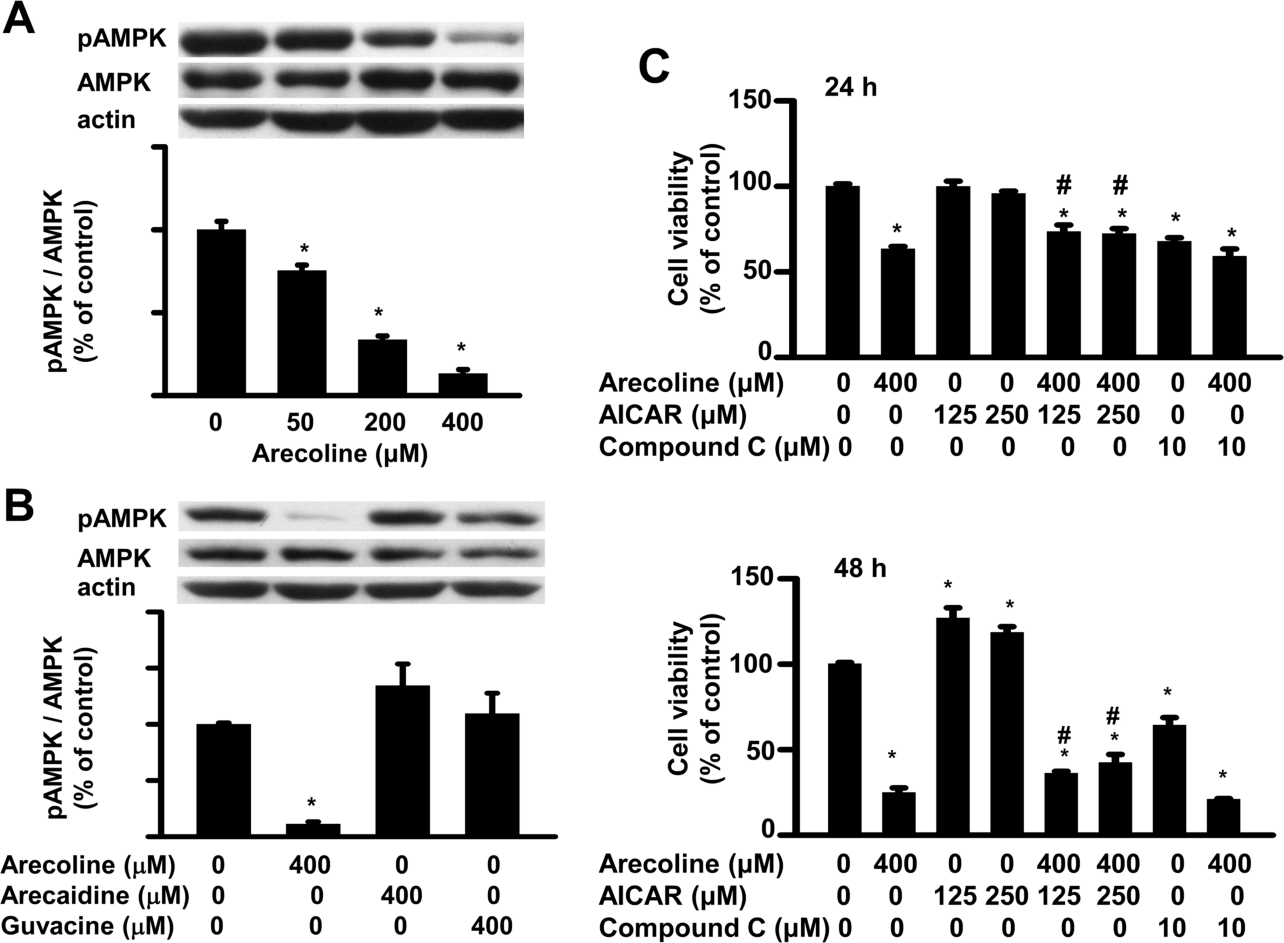
The effect of arecoline on cell viability in 3T3-L1 preadipocyte was dependent on the AMP-activated protein kinase (AMPK) pathway. (A) Arecoline dose-dependently reduced protein amounts of phospho-AMPK (pAMPK) but not the total amount of AMPK protein. (B) An alkaloid-specific effect of betel nut on AMPK phosphorylation was observed after 24 h of 400-μM treatment. (C) The AMPK activator AICAR at doses of 125 and 250 μM antagonized the arecoline-reduced cell viability of preadipocytes, while its inhibitor compound C (10 μM) alone inhibited cell viability but did not block arecoline-reduced cell viability. Total AMPK was measured by Western blot analysis and then expressed after normalization to actin, whereas pAMPK was normalized to its total AMPK. Data are expressed as the mean ± SEM from triplicate determinations. *, *p* < 0.05 *vs*. the control; #, *p* < 0.05 arecoline *vs*. arecoline + AICAR.

Preadipocyte growth can be regulated by AMPK [21]. We investigated which AMPK signaling molecule arecoline acted on to regulate 3T3-L1 preadipocyte viability. First, we examined whether arecoline affected the levels and activity of AMPK protein (Fig 4). The activity of AMPK was assessed by changes in the amount of the threonine^172^-phosphorylated form of AMPK [23]. We found that 50~400 μM arecoline for 24 h did not alter the total amount of AMPK protein, but it decreased the phosphorylation of AMPK in a dose-dependent manner (Fig 4A). At 400 μM, neither arecaidine nor guvacine altered the amounts of total AMPK protein or the phosphorylation of AMPK after 24 h of treatment (Fig 4B).

Next, we assessed whether the AMPK activator, AICAR, could affect the arecoline suppression of 3T3-L1 preadipocyte viability (Fig 4C). We found that 125 and 250 μM AICAR for 48 h increased preadipocyte viability. When added in the presence of arecoline, it significantly reduced arecoline-induced suppression of cell viability. On the other hand, the specific inhibitor of AMPK, compound C, significantly reduced 3T3-L1 preadipocyte viability after 24 or 48 h of treatment. Moreover, in the presence of arecoline, it did not prevent arecoline-induced suppression of cell viability.

AMPK is an essential part of the signaling cascade regulated by cell surface receptors in response to environmental stimulation [31]. For example, AMPK mediates arecoline regulation of oral cancer cell growth [24], and AMPK modulates IGF-I mitogenic signaling in 3T3-L1 preadipocytes [21]. We found that AMPK activation via AICAR antagonized arecoline suppression of cell viability at 24 and 48 h. This result suggested that arecoline regulated preadipocyte viability by inhibiting AMPK function. This interpretation was supported by the findings that arecoline reduced the levels of phosphorylated AMPK in 3T3-L1 preadipocytes, and the specific inhibitor of AMPK (compound C) alone could significantly reduce 3T3-L1 preadipocyte viability at 24 or 48 h. This AMPK-dependent effect of arecoline on 3T3-L1 preadipocytes was consistent with results from a previous study on human oral fibroblasts [24].

### Arecoline effects on preadipocyte growth depended on ROS

**Fig 5:**
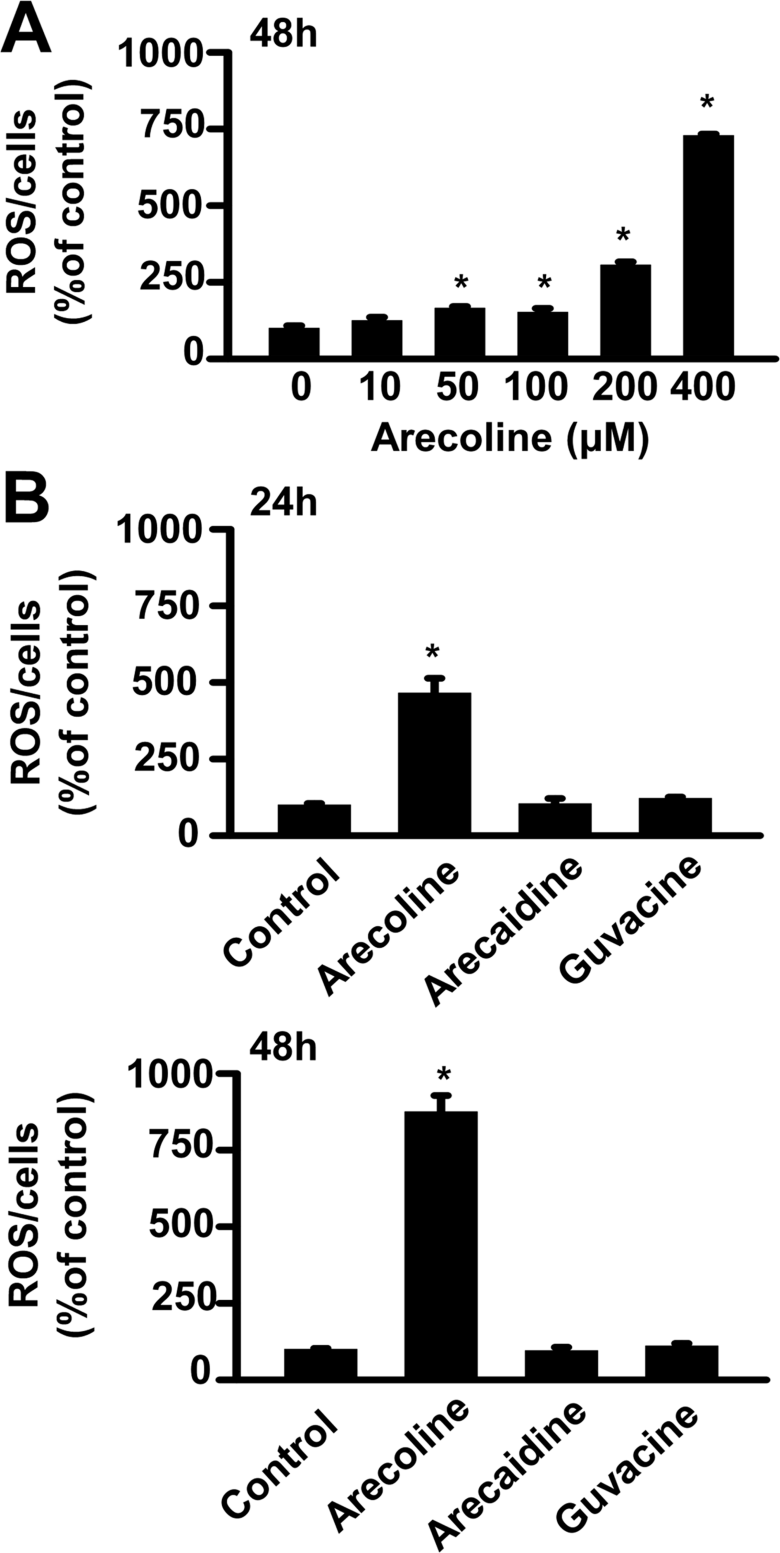
The alkaloid-dependent effect of betel nut on the production of reactive oxygen species (ROS). (A) A dose-dependent effect of arecoline was observed after 48 h of treatment. (B) Arecoline, but not arecaidine or guvacine, induced ROS production after 24 and 48 h of 400-μM treatment. ROS production was measured by the 2’,7’-dichlorofluorescein diacetate (DCFDA) method. *, *p* < 0.05 *vs*. the control.

**Fig 6:**
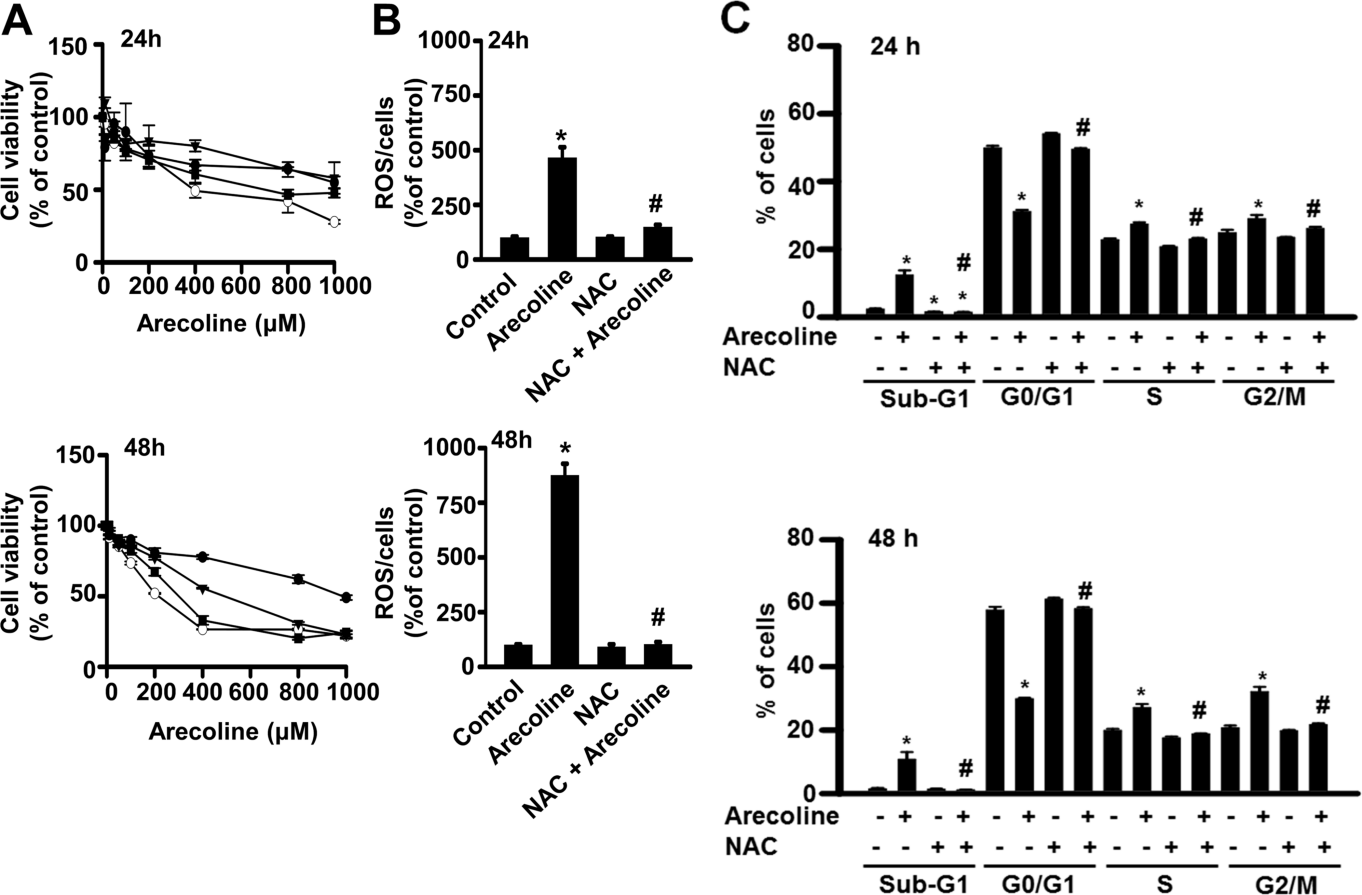
***N*-acetylcysteine (NAC) blocked arecoline-altered cell viability (A), reactive oxygen species (ROS) production (B), and the four different phases of the cell cycle (C) in 3T3-L1 preadipocytes.** 3T3-L1 preadipocytes were pretreated with NAC for 1 h and then exposed to betel nut alkaloid. After 24 and 48 h of treatment, cell viability, ROS production, and phases of the cell cycle were examined by the MTT, 2’,7’-dichlorofluorescein diacetate incorporation, and flow cytometry, respectively. In A, the NAC dosages were 0, 1.25, 2.5 and 5 mM for open circle, open square, open triangle, and closed circle, respectively, while in B and C, 5 mM NAC was used. Data are expressed as the mean ± SEM from triplicate determinations. In (A), statistical significances are not shown for clarity. *, *p* < 0.05 *vs*. the control; #, *p* < 0.05 arecoline *vs*. NAC + arecoline.

**Fig 7:**
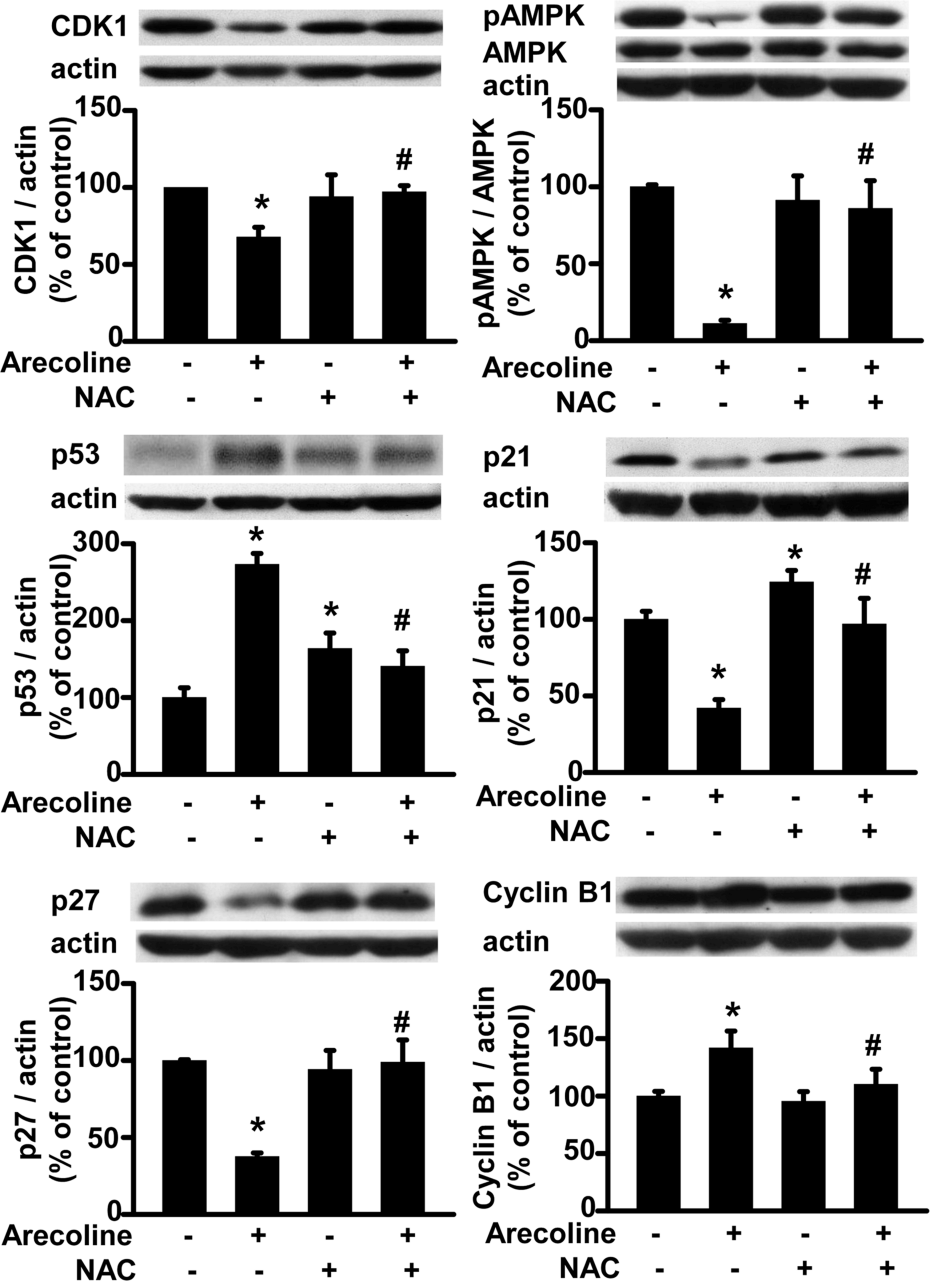
*N*-acetylcysteine (NAC) blocked the arecoline-altered cell cycle-controlling pathway proteins, such as cyclin-dependent kinase 1 (CDK1), p53, p21, p27, and cyclin B1, as well as the phosphorylation of AMPK protein in 3T3-L1 preadipocytes. Cells were pretreated with NAC for 1 h and then exposed to betel nut alkaloids. After 24 h of treatment, proteins were examined by Western blot analysis and then expressed after normalization to actin, whereas pAMPK was normalized to its total AMPK. Data are expressed as the mean ± SEM from triplicate determinations; each determination was pooled from four 10-cm culture plates. *, *p* < 0.05 *vs*. the control; #, *p* < 0.05 arecoline *vs*. NAC + arecoline.

Although the arecoline growth-inhibitory effect in non-fat cells was reported to be ROS-dependent [13–16], preadipocytes have not been investigated. Accordingly, we examined whether the arecoline effects on cell viability, growth arrest, and cell cycle-control molecules were dependent on the ROS pathway in 3T3-L1 preadipocytes (Figs 5, 6 and 7). First, we analyzed whether BNAs induced ROS production in 3T3-L1 preadipocytes (Fig 5). Indeed, we found that arecoline (Fig 5A and 5B), but not arecaidine or guvacine (Fig 5B), significantly increased ROS production in preadipocytes, in a dose- and time-dependent manner.

Pretreatment of 3T3-L1 preadipocytes with the antioxidant, NAC, dose-dependently prevented arecoline-induced suppression of cell viability (Fig 6A). At 5 mM, NAC pretreatment also blocked the time-dependent effect of 400 μM arecoline. In addition, 5 mM NAC pretreatment blocked arecoline-induced stimulation of ROS production (Fig 6B). Moreover, NAC pretreatment blocked arecoline-induced suppression of G0/G1 growth arrest and arecoline-induced increases in the sub-G0, S, and G2/M phases, after 24 or 48 h (Fig 6C).

Next, we examined whether NAC affected the arecoline-induced alterations in AMPK phosphorylation and cell cycle-control proteins (Fig 7). NAC pretreatment antagonized the 24-h arecoline effects on AMPK protein phosphorylation and cell cycle-control protein expression (suppression of CDK1, p21, and p27 and stimulation of p53 and cyclin B1 expression). Either alone or in combination with arecoline, NAC could not alter the total AMPK (Fig 7) or CDK2 protein levels (data not shown).

Our study provided evidence that the antioxidant, NAC, prevented several arecoline-induced effects, including its stimulation of ROS production and its suppression of cell viability and AMPK activity. In addition, NAC blocked arecoline-induced dysregulation of the cell cycle by reversing arecoline-altered levels of CDK1, p21, p27, p53, and cyclin B1 proteins. These observations suggested that the inhibitory effect of arecoline on cell viability was mediated by a pathway that required induction of ROS production. It was previously shown that arecoline could produce different metabolites and free radical groups, including arecoline N-oxide, arecoline N-oxide mercapturic acid, nitrosamines, 3-methylnitrosaminopropionate, and N-nitrosoguvacoline [32–33]. However, we did not investigate whether that mechanism could explain the ROS-dependent effects of arecoline on preadipocyte viability in this study.

Betel nut alkaloids have numerous biological activities with various biological effects [34–35]. Most studies reported that arecoline was more active than the other alkaloids. Our findings supported that observation. In 3T3-L1 preadipocytes, at the same doses and durations, arecoline was generally more effective than arecaidine and guvacine in inhibiting cell viability; changing the distributions of cells in different phases of the cell cycle; modulating the levels of CDK1, cyclin B1, p27, and phosphorylated AMPK proteins; and increasing the level of ROS production.

These alkaloid-specific effects of betel nut indicated that arecoline may act differently from arecaidine and guvacine in regulating preadipocyte viability. These three alkaloids have unique structures [35]. Arecoline, but not arecaidine or guvacine, contains a methyl ester group on its *N*-containing aromatic ring. This methyl ester group may be important for conformational flexibility and interactions with other molecules. Although, like arecoline, arecaidine and guvacine could increase p53 and decrease p21 protein levels, their potencies were lower than that of arecoline. This lack of potency might explain the small effects of both arecaidine and guvacine on preadipocyte growth. Alternatively, preadipocyte viability might be mainly regulated by p27, cyclin B1 and CDK1, but not p53 or p21, because arecoline, but not arecaidine or guvacine, significantly reduced p27 and CDK1 and increased cyclin B1.

Of note, fresh betel nuts contain 0.3–0.63% arecoline, 0.31–0.66% arecaidine, and 0.19–0.72% guvacine [36]. Thus, it might be worth exploring arecaidine and guvacine at higher concentrations than those used in this experiment to determine whether they have effects on preadipocyte growth and signaling.

Mechanistic studies of betel nut arecoline have shown that its effects depended on the cell type [13–16,23–24]. This notion was supported by findings that arecoline induced G2-phase arrest in 3T3-L1 preadipocytes, basal cell carcinoma cells, and oral fibroblasts, but arecoline induced G1-phase arrest in hepatocytes and keratinocytes [13–14,16,23–24]. A potential explanation could be that the sensitivity to betel nut arecoline varies among normal, transformed, and cancer cells. Alternatively, the differences may be due to the different cell culture techniques and assay methods employed.

**Fig 8:**
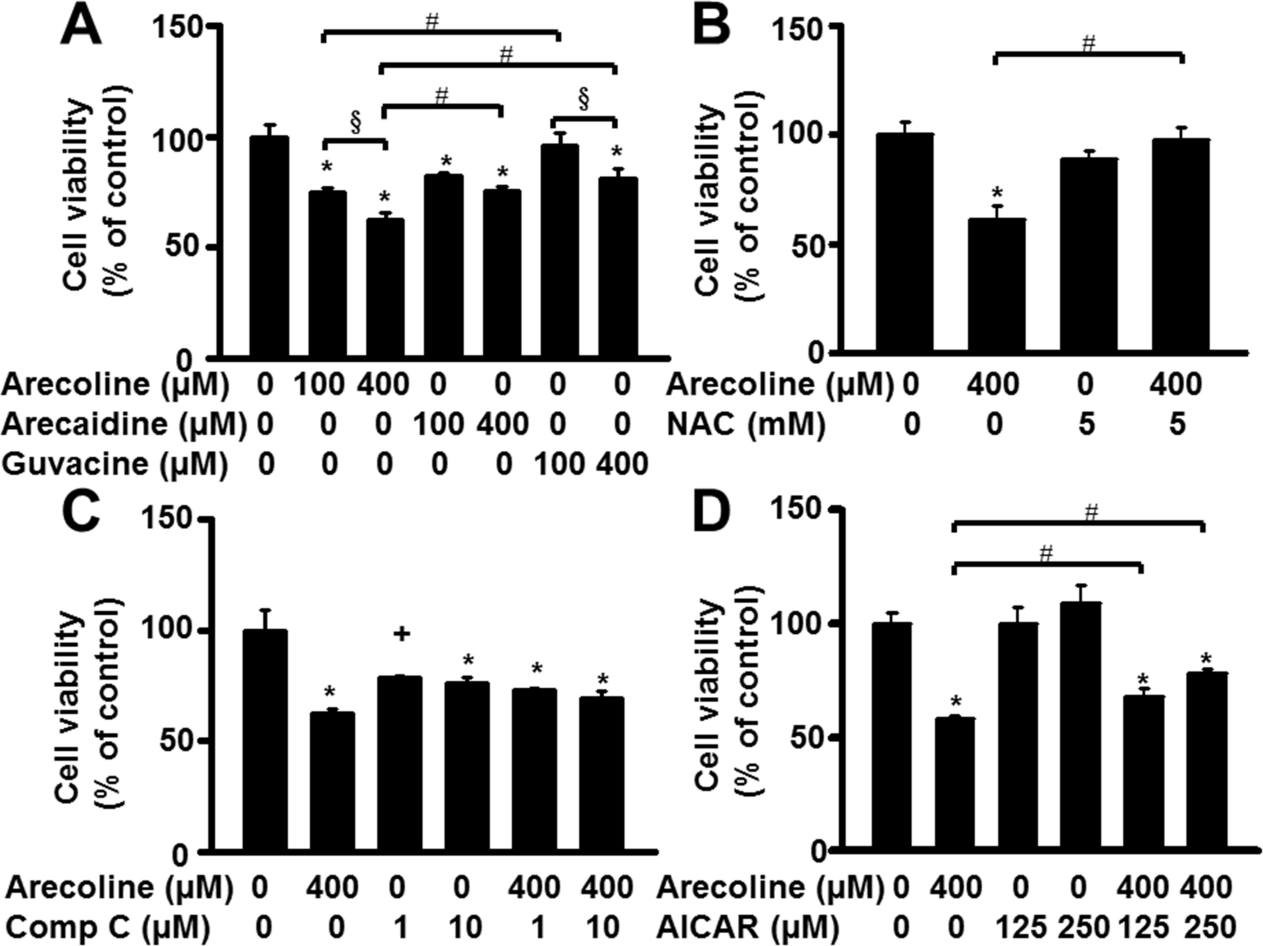
Betel nut alkaloids reduced cell viability of primary preadipocytes isolated from the epididymal adipose tissues of male mice after 48 h of treatment, and the effect of arecoline depended on AMPK and ROS pathways. Data are expressed as the mean ± SEM from triplicate determinations. +, *p* < 0.1 *vs*. control; *, *p* < 0.05 *vs*. control; #. *p* < 0.05, arecoline *vs*. arecaidine, arecoline *vs*. guvacine, arecoline *vs*. NAC + arecoline, or arecoline *vs*. AICAR + arecoline (bracket); §, *p* < 0.05, 100 μM *vs*. 400 μM.

We also found that 48 h of 100 and 400 μM arecoline inhibited the viability of primary preadipocytes (Fig 8A) isolated from epididymal adipose tissues of male mice, according to a collagenase digestion method [37]. Again, we found that the inhibitory effect of arecoline was greater than that of arecaidine or guvacine. In addition, both NAC and AICAR, but not compound C, prevented arecoline-induced suppression of cell viability in primary preadipocytes (Fig 8B, 8C and 8D). Interestingly, 100 and 400 μM arecaidine and 400 μM guvacine could significantly inhibit the viability of primary preadipocytes (Fig 8A), but not 3T3-L1 preadipocytes (Fig 1). A possible explanation for this discrepancy might be that the various growth factors, nutrients, and heterogeneous cells present in primary cell cultures might affect their viability in response to arecaidine and guvacine. Unfortunately, we could not isolate primary adipocytes in the present study, due to interference from excessive lipid accumulation. Thus, further studies are needed to investigate whether the same arecoline-mediated cytotoxic mechanism occurs in primary adipocytes.

**Fig 9:**
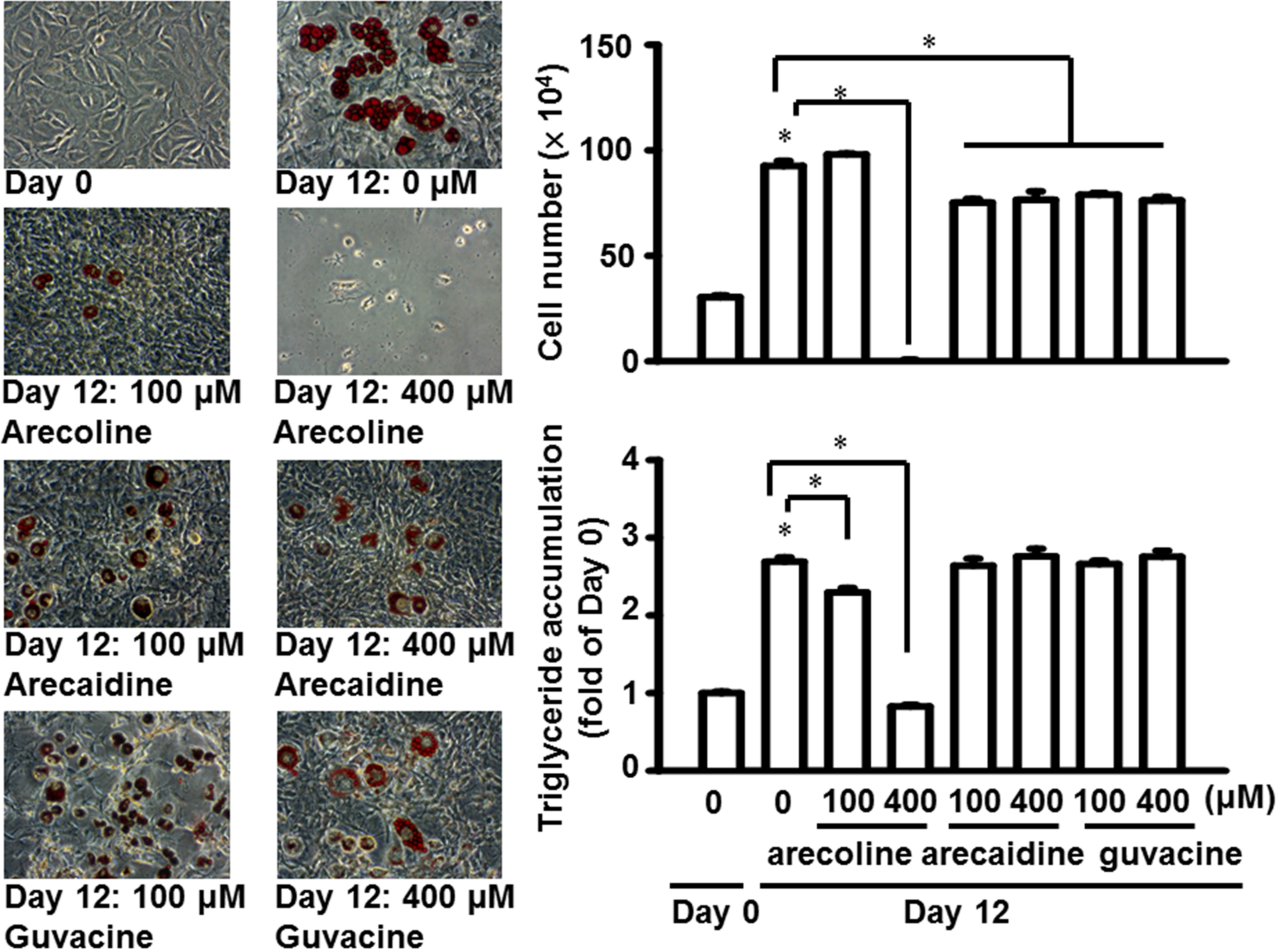
Arecoline, arecaidine, and guvacine differentially affected cell number and total triglyceride accumulation during the 12-day period of adipogenic differentiation of 3T3-L1 adipocytes. Data are expressed as the mean ± SEM from triplicate determinations. *, *p* < 0.05, Day 8 vs. Day 0 with no alkaloid treatment, or alkaloid vs control on Day 8 (bracket).

Arecoline was previously shown to regulate other adipocyte functions [10–12]. *In vitro*, arecoline inhibited adipogenic differentiation; the expression of fatty acid synthase and HMG-CoA reductase genes; and insulin-induced glucose uptake and insulin signaling; in addition, it induced lipolysis in differentiated adipocytes [10–12]. In the present study, we found that arecoline, at 400, but not 100 μM, reduced the number of 3T3-L1 cells during a 12-day period of adipogenic differentiation (Fig 9) [37]. Moreover, both arecoline concentrations significantly reduced the total triglyceride accumulation during adipogenic differentiation. Neither arecaidine nor guvacine at 100 or 400 μM altered total triglyceride accumulation under the same culture conditions, although both alkaloids, at both concentrations, slightly altered the cell numbers. This result indicated that different alkaloids had different effects on the adipogenic differentiation of fat cells. Further investigation is required to determine whether arecoline, arecaidine, or guvacine will exhibit effects on preadipocyte differentiation *in vivo*.

## Conclusions

**Fig 10:**
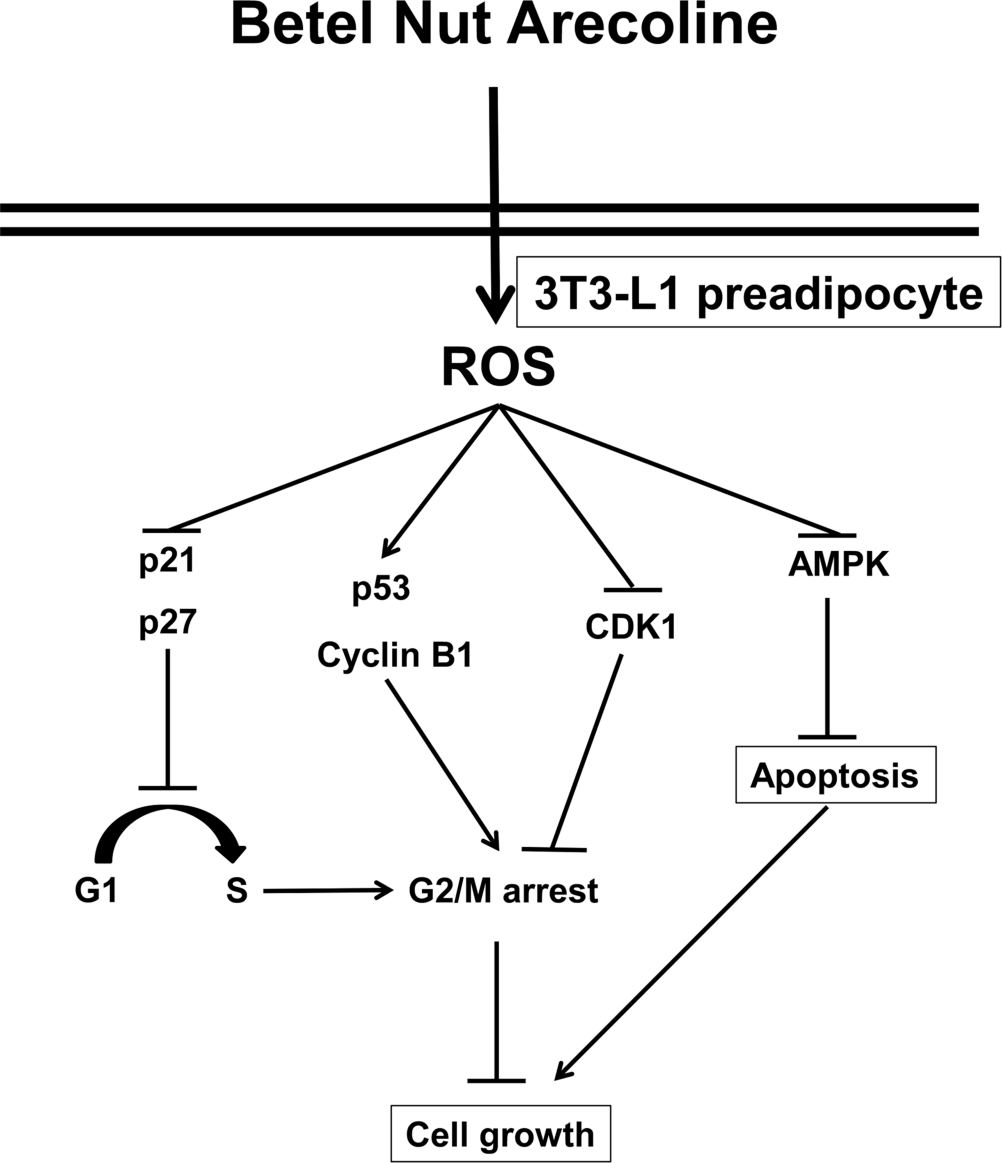
A proposed mechanism of the action of betel nut arecoline on cell growth in 3T3-L1 preadipocytes. The arecoline signaling was dependent on the AMPK, reactive oxygen species, and, possibly, cell cycle-controlling protein pathways.

We demonstrated that the effects of arecoline on 3T3-L1 preadipocyte growth were time- and dose-dependent, and they were most likely mediated through dysregulation of the cell cycle (Fig 10). The effects involved the inactivation of AMPK activity and the inhibition of particular members of the CDK family (e.g., CDK1 protein) and the CKI pathway (e.g., p27 protein) through an intracellular ROS pathway. In general, arecoline was more effective than two other structurally related betel nut alkaloids, arecaidine and guvacine, in altering preadipocyte signaling and adipogenic differentiation. Our elucidation of the mechanism underlying arecoline effects on preadipocytes may improve our understanding of how arecoline and arecoline-containing betel nut extracts affect body weight, fat cells, and metabolic syndrome [3–12]. Both the acetylcholine receptor and the gamma-butyric acid receptor function as arecoline receptors [34–35]. Further studies are needed to determine whether those receptors might be involved in the observed effects of arecoline on fat cells.
